# Opium Antidotes

**Published:** 1877-10

**Authors:** 


### Opium Antidotes.

Dr. J. B. Mattison, of Brooklyn, has done good service in exposing some of the numerous *opium antidote quacks,* who are advertising largely. He shows that all their antidotes are composed of morphia in gradated doses.

